# Supplementary material for: Selenoprotein W ensures physiological bone remodeling by preventing hyperactivity of osteoclasts
Source: Nat Commun. 2021 Apr 15;12:2258. doi: 10.1038/s41467-021-22565-7 (PMC8050258; doi:10.1038/s41467-021-22565-7)
Supplement: Supplementary file 1 — Supplementary Information [file 41467_2021_22565_MOESM1_ESM.docx]

**Supplementary Table 1. Primer lists used in RT-PCR and ChIP assay**

| Semiquantitative RT-PCR | | | | | | |
| --- | --- | --- | --- | --- | --- | --- |
| Gene | | Sense (5′→3′) | Anti-sense (5′→3′) | | | |
| *NFATc1*  *F4/80*  *SELENOW*  *Acp5*  *OSCAR*  *c-Fos*  *c-Src*  *Ctsk*  *Alp*  *Spp1*  *HPRT*  *GAPDH* | | CTCGAAAGACAGCACTGGAGCAT  GATGAATTCCCGTGTTGTTGGT  CGACGTGCAGCTATGGCGCTC  ACTTCCCCAGCCCTTACTACC  AGATGGACAGAGAAGCTGGGATCC  CTGGTGCAGCCCACTCTGGTC  CCAGGCTGAGGAGTGGTACT  CTTGTGGACTGTGTGACT  GGCCCTCTCCAAGACATATAAC  ACACTTTCACTCCAATCGT  GTAATGATCAGTCAACGGGGGAC  CAAGGCTGTGGGCAAGGTCA | CGGCTGCCTTCCGTCTCATAG  ACATCAGTGTTCCAGGAGACACA  GCAGGGCAGGCTGCGTGCACG  TCAGCACATAGCCCACACCG  TTAGGAGCCAGCCAGATGGCTCAG  CTTTCAGCAGATTGGCAATCTC  CAGCTTGCGGATCTTGTAGT  AACACTGCATGGTTCACA  TGATGAGATCCAGGCCATCTA  TGCCCTTTCCGTTGTTGT  CCAGCAAGCTTGCAACCTTAACCA  AGGTGGAAGAGTGGGAGTTGCTG | |  |  |
| ChIP assay | | | | | | |
| Gene | Sense (5′→3′) | | | Anti-sense (5′→3′) | |  |
| *NFATc1*  *NF-κB* | CCGGGACGCCCATGCAATCTGTTAGTAATT  GTGCCTCCCCAATGTGCTAA | | | GCGGGTGCCCTGAGAAAGCTACTCTCCCTT  AAAGAAACTGCCCTCCCTCTC | |  |

**
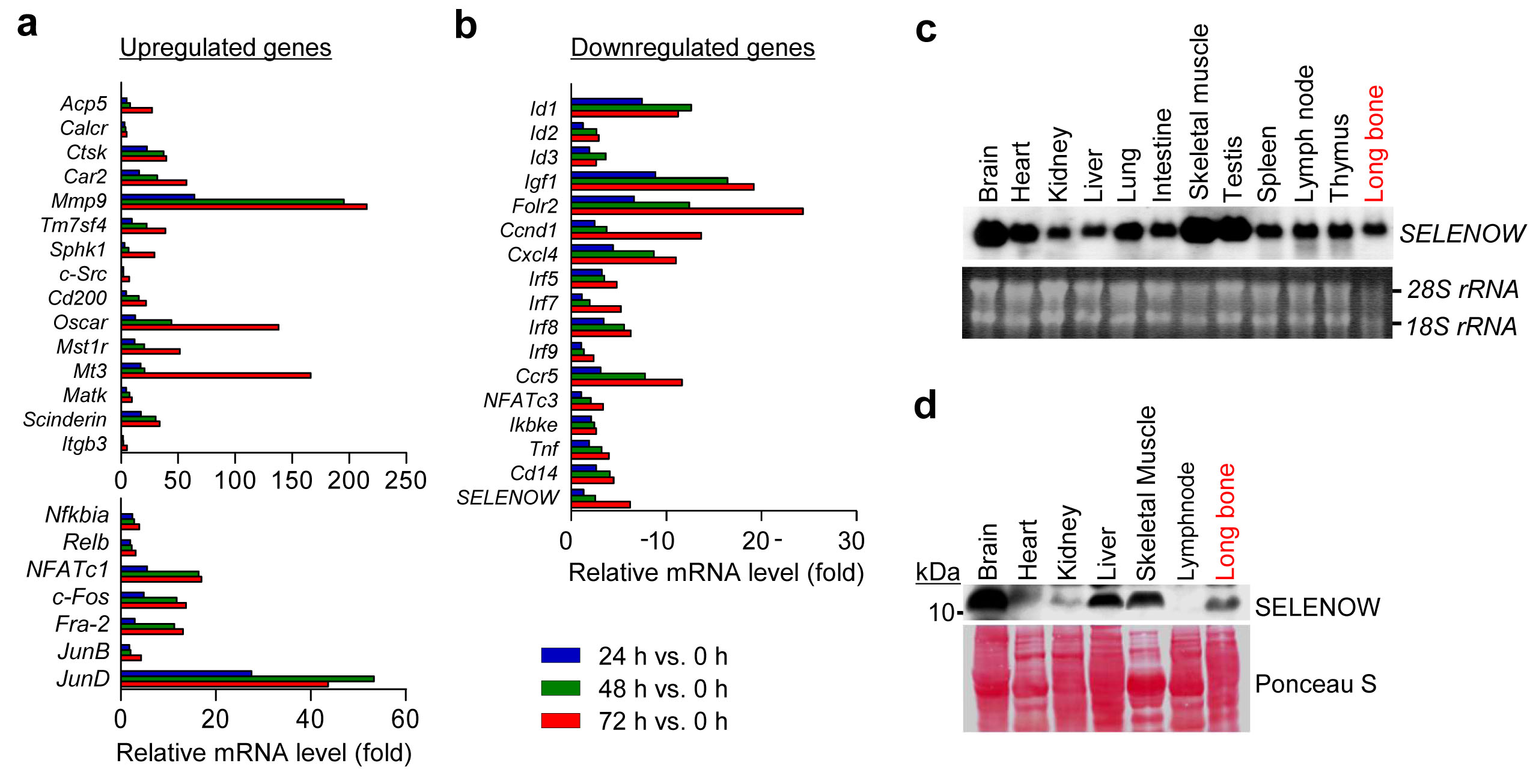
**

**Supplementary Figure 1** Gene expression profiles during RANKL-induced osteoclastogenesis and tissue-specific distribution of *SELENOW*. **a, b** GeneChip analysis of genes upregulated (**a**) and downregulated (**b**) during osteoclast differentiation. Bone marrow-derived osteoclast precursors were stimulated with RANKL in the absence or presence of M-CSF for 24, 48, or 72 h. RNA was extracted and analysed with a GeneChip array. The level of representative genes showing a 3-fold or greater change is depicted as fold induction or repression relative to control osteoclast precursors treated with M-CSF alone (*n* = 3). **c, d** Tissue-specific expression patterns of SELENOW. Total RNA and whole protein extracts were isolated from various tissues of C57BL6 mice. *SELENOW* mRNA levels was examined by northern blotting using [^32^P]dCTP-labelled cDNA probes (**c**). Acridine orange-stained 18S and 28S rRNA bands served as a loading control. SELENOW protein levels were evaluated by immunoblotting analysis with a SELENOW-specific antibody (**d**). Ponceau S-stained nitrocellulose membrane before antibody probing served as a protein loading control. Images are representative of three independent experiments.


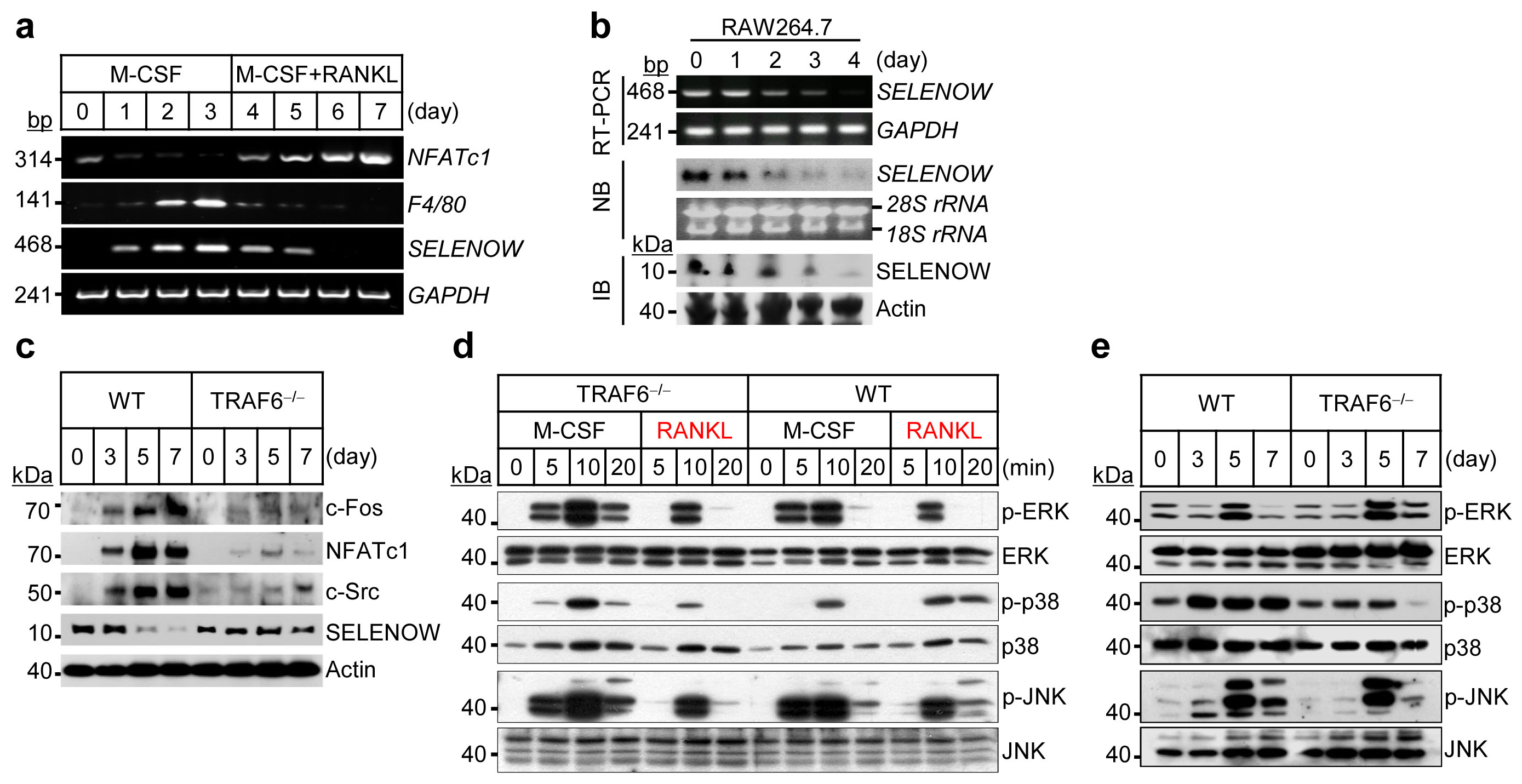


**Supplementary Figure 2** SELENOW is upregulated during macrophage differentiation and downregulated via TRAF6 during RANKL-induced osteoclast differentiation. **a** Change in *SELENOW* gene expression in M-CSF-induced differentiation of macrophages and osteoclast differentiation induced by treatment with M-CSF and RANKL. *SELENOW* mRNA levels were analysed by RT-PCR. F4/80 and NFATc1 served as markers of macrophages and osteoclasts, respectively. **b** Murine RAW264.7 macrophages were induced to differentiate into osteoclasts in the presence of RANKL for indicated times, and *SELENOW* expression was assessed by RT-PCR, northern blotting (NB), and immunoblotting (IB). **c** TRAF6-deficient (TRAF6^−/−^) embryonic liver-derived osteoclast precursors failed to induce SELENOW downregulation during RANKL-induced osteoclast differentiation in the indicated times, as shown by immunoblot analysis with antibodies specific to osteoclastogenic markers (c-Fos, NFATc1, and c-Src) and SELENOW. **d, e** TRAF6-dependent p38 activation. TRAF6^−/−^ osteoclast precursors derived from embryonic liver failed to activate p38 signalling in the immediate (**d**) and delayed response (**e**) to RANKL, while ERK and JNK activation were unaffected, as demonstrated by immunoblotting. WT, wild type. Images are representative of three independent experiments.


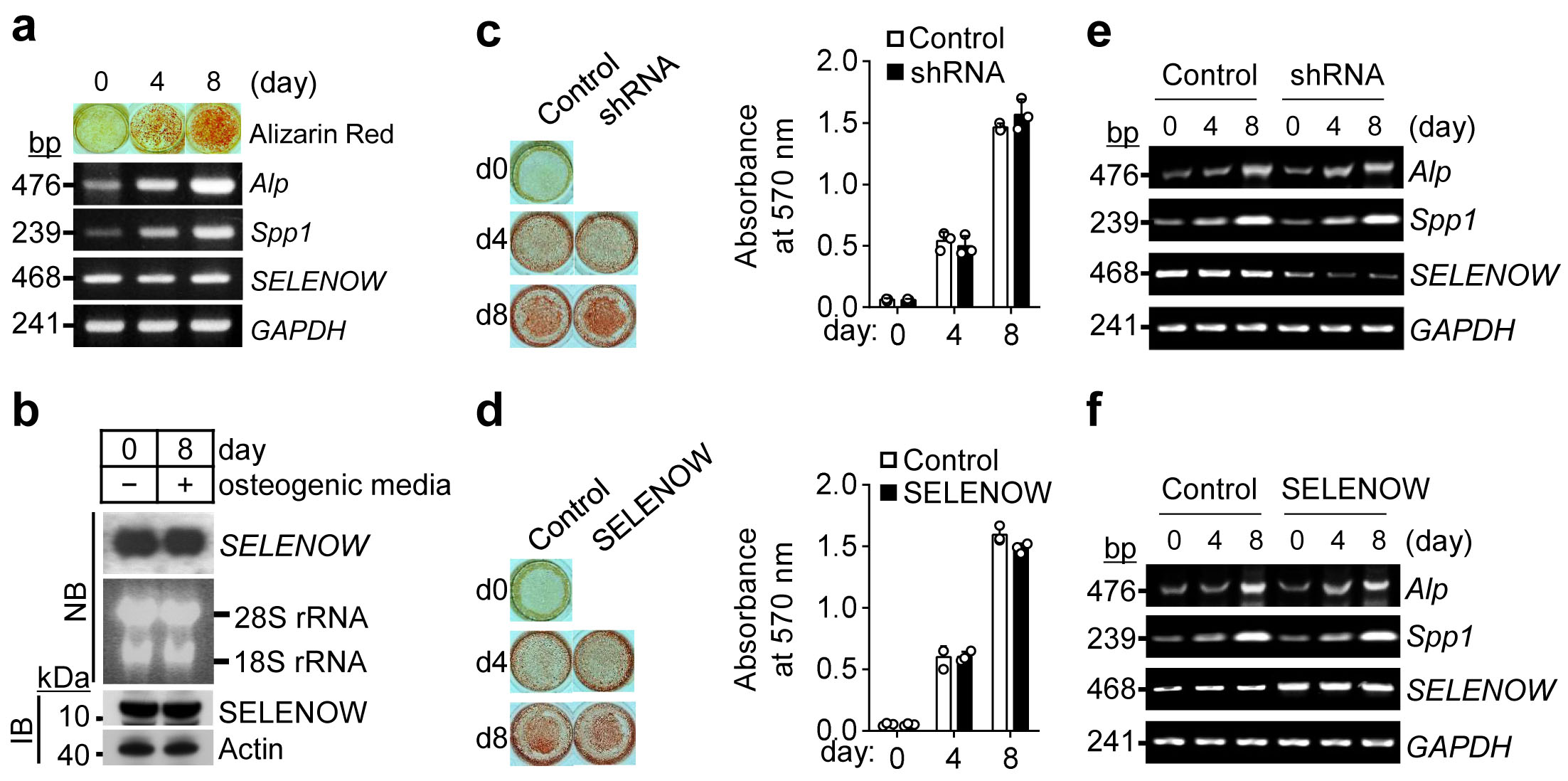


**Supplementary Figure 3** Effect of *SELENOW* deficiency and overexpression on osteoblast differentiation. **a, b** Change in SELENOW expression during osteoblast differentiation. Calvarial primary osteoblasts were cultured in osteogenic medium containing 100 μg/ml ascorbic acid and 10 mM β-glycerol phosphate for 8 days, and the extent of osteoblast differentiation was visualised by Alizarin Red S staining; expression of osteoblast marker genes (*Alp* and *Spp1*) and SELENOW was determined by RT-PCR (**a**), northern blotting (NB), or immunoblotting (IB) (**b**). **c, d** Regulation of osteoblast differentiation by SELENOW. The expression of *SELENOW* in osteoblasts was suppressed by lentiviral transduction of shRNA and induced by constitutive expression of *SELENOW* from a retroviral vector; cells were induced to differentiate in osteogenic medium for indicated times. Osteoblast differentiation was visualised by Alizarin Red S staining (**c**, **d**, left panels) and quantified by measuring the absorbance of extracts from stained samples at 570 nm (**c**, **d**, right panels, *n* = 3). **e, f** To evaluate the extent of osteoblast differentiation, the mRNA levels of osteoblast marker genes (*Alp* and *Spp1*) and *SELENOW* were evaluated by RT-PCR. Data represent mean ± SD of triplicate samples. Images are representative of three independent experiments.


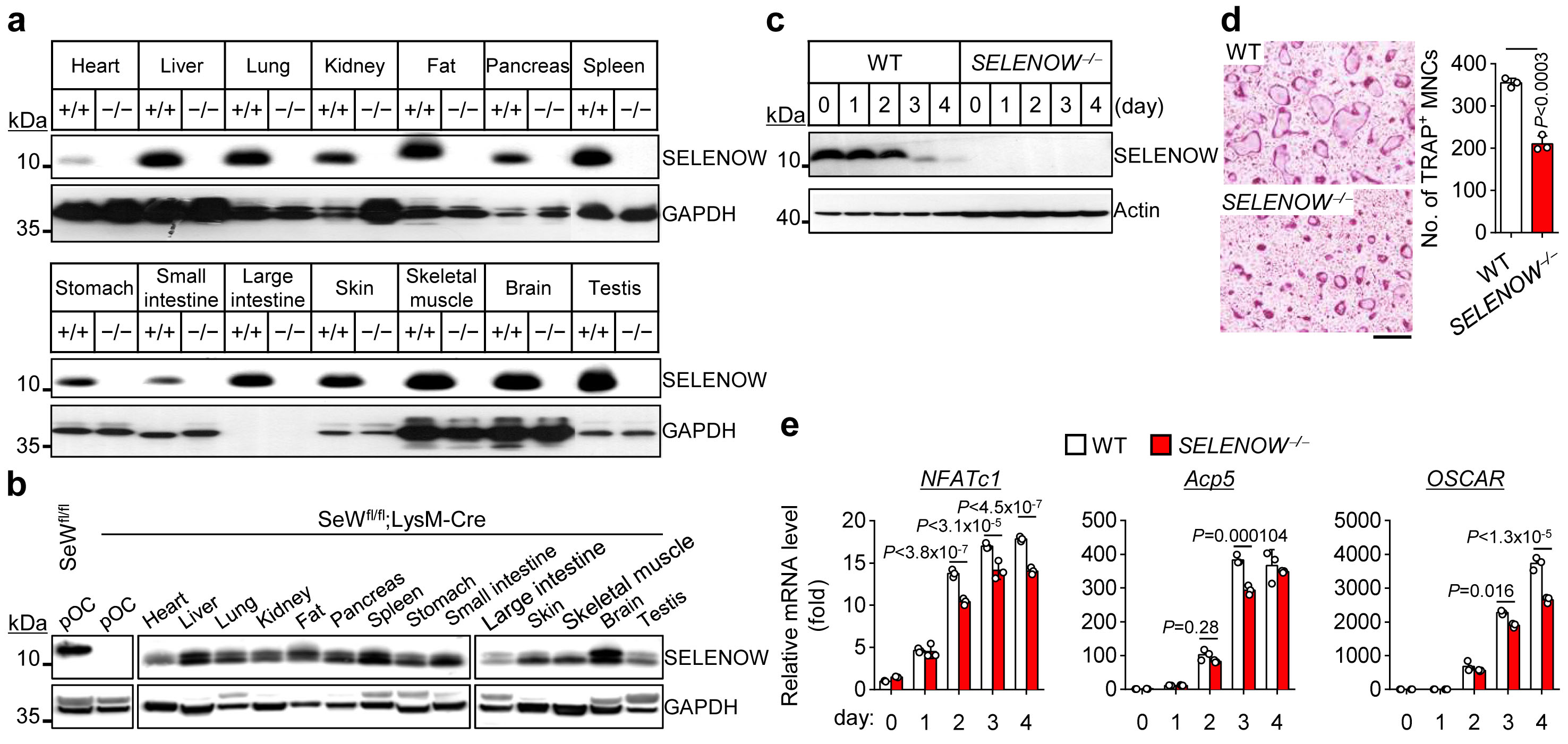


**Supplementary Figure 4** SELENOW expression levels in SELENOW-deficient male mice. **a**, **b** Whole lysates prepared from various tissues of wild-type (WT) littermates (+/+) and *SELENOW*^−/−^ mice (−/−) or WT littermates (SeW^fl/fl^) and osteoclast-specific SELENOW knockout mice (SeW^fl/fl^; LysM-Cre) were subjected to immunoblot with specific antibody to SELENOW; GAPDH used as a loading control. pOC; osteoclast precursors. **c** Expression of SELENOW during differentiation of *SELENOW*^−/−^ osteoclast precursors. **d** Defective osteoclast formation in *SELENOW*^−/−^ osteoclast precursors (*n* = 3). Scale bar, 100 μm. **e** Downregulation of osteoclast marker genes (*NFATc1*, *Acp5,* and *OSCAR*) during the differentiation of *SELENOW*^−/−^ osteoclast precursors. Total RNA was collected on the indicated days and analysed using qPCR (*n* = 3). Data represent mean ± SD of triplicate samples. Statistical significance was determined by Student’s two-tailed t test (**d**). Images are representative of three independent experiments.


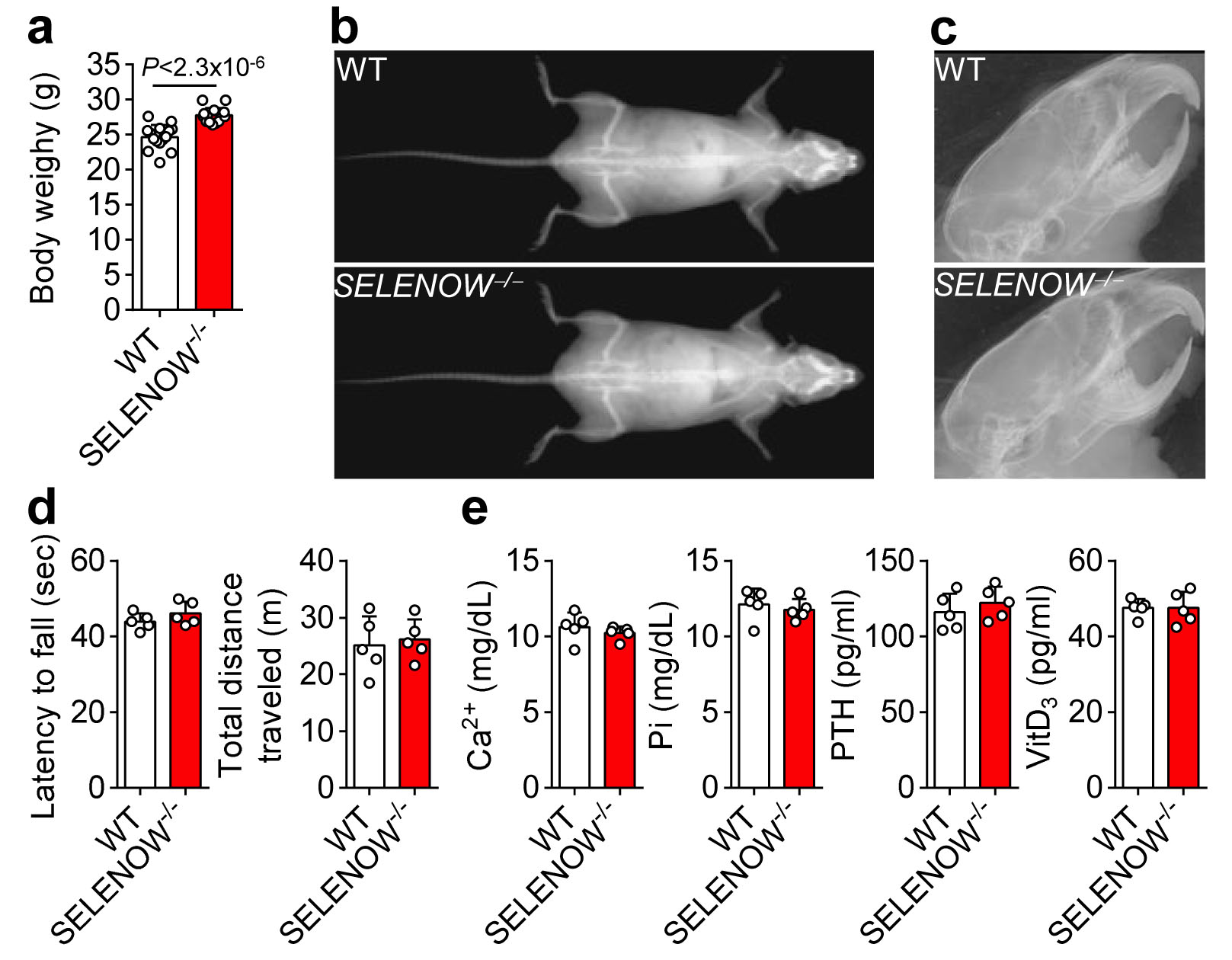


**Supplementary Figure 5** Change in body and bone appearance of *SELENOW*^−/−^ male mice at 10 weeks. **a** Comparison of body weight in WT and *SELENOW*^−/−^ male mice (*n* = 15). **b** X-ray appearance for whole body using a Dual-Energy X-ray Absorptiometry (InAnalyzer, Medikors, Korea). **c** Examination of tooth eruption using a soft X-ray radiography. **d** Locomotor activity. Latency to fall on the rotarod with 3 cm rod diameter was recorded (left panel, *n* = 5); total distance travelled on an open field was measured using the SAMRT automated video tracking (right panel, *n* = 5). **e** Analysis of serum bone-related parameters [Ca^2+^, Pi, parathyroid hormone (PTH), vitamin D_3_ (VitD_3_)] from WT littermates and *SELENOW*^−/−^mice using ELISA kit (according to manufacturer instructions). Data represent mean ± SD (n = 5). Statistical significance was determined by Student’s two-tailed t test (**a**).

**
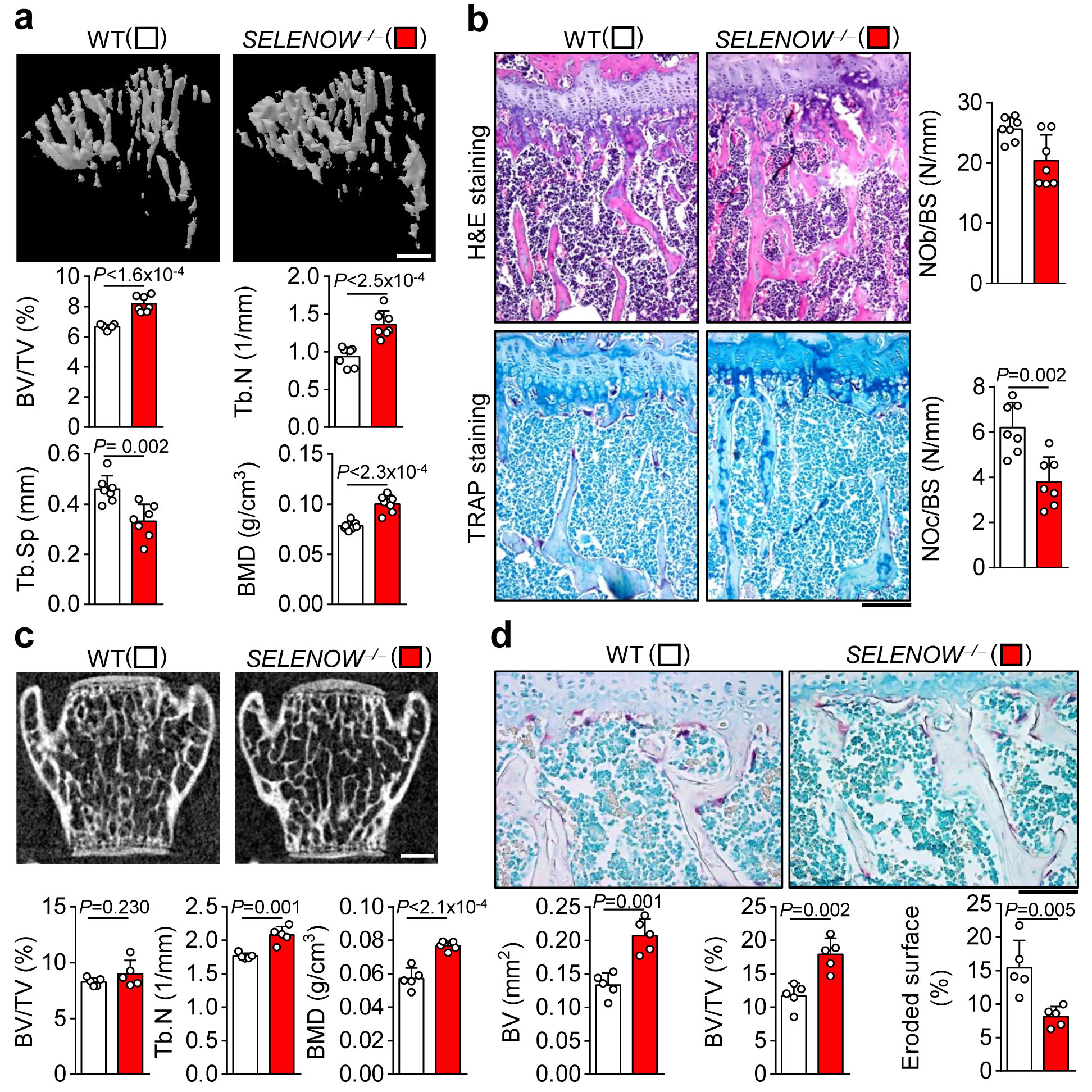
**

**Supplementary Figure 6** Increased bone mass phenotypes in *SELENOW*^−/−^ mice. **a** μCT analysis of proximal tibiae from wild-type (WT) female littermates and age/sex-matched *SELENOW*^−/−^ mice at 10 weeks. BV/TV, trabecular bone volume per tissue volume; Tb.N, trabecular bone number; Tb.Sp, trabecular separation; BMD, bone mineral density. Scale bar, 0.5 mm. **b** Defective osteoclast formation in trabecular bone surface of *SELENOW*^−/−^female mice; NOb/BS from H&E-stained sections (upper panels; NOc/BS from TRAP-stained sections (lower panels); Scale bar, 100 μm. **c** μCT analysis of trabecular bone in the lumbar vertebra (L4) from WT male littermates and *SELENOW*^−/−^ mice. BV/TV, trabecular bone volume per tissue volume; Tb.N, trabecular bone number. **d** Bone histomorphometric analysis for BV, BV/TV and eroded bone surface from TRAP-stained lumbar trabecular bone sections; Scale bar, 100 μm. Data represent mean ± SD (*n* = 7 mice per group in **a** and **b**, *n* = 5 mice per group in **c** and **d**). Statistical significance was determined by Student’s two-tailed t test.


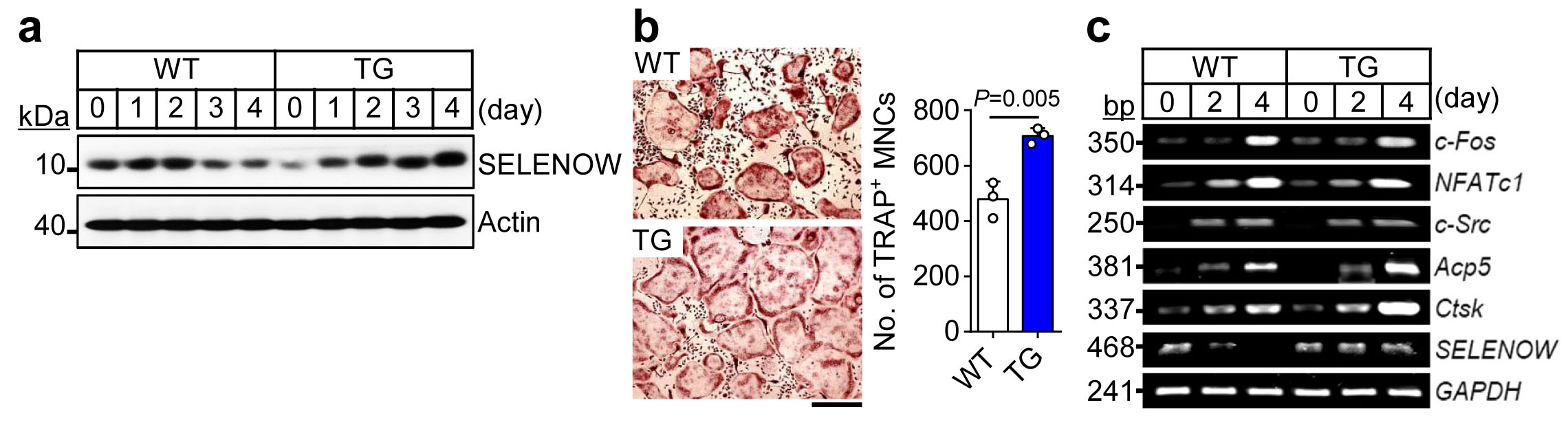


**Supplementary Figure 7** Accelerated osteoclast formation in osteoclast precursors from *SELENOW*-expressing transgenic mice. **a** SELENOW expression in whole extracts during osteoclast differentiation of osteoclast precursors from wild-type (WT) littermates and transgenic mice (TG) was evaluated using immunoblotting with an anti-SELENOW antibody. **b** Increased osteoclast formation in *SELENOW*-overexpressing osteoclast precursors; Scale bar, 100 μm. **c** mRNA levels of the osteoclast-specific marker genes *c-Fos*, *NFATc1*, *c-Src*, *Acp5*, and *Ctsk* and of *SELENOW* were determined by RT-PCR (*n* = 3). Data represent mean ± SD of triplicate samples. Statistical significance was determined by Student’s two-tailed t test. Images are representative of three independent experiments.


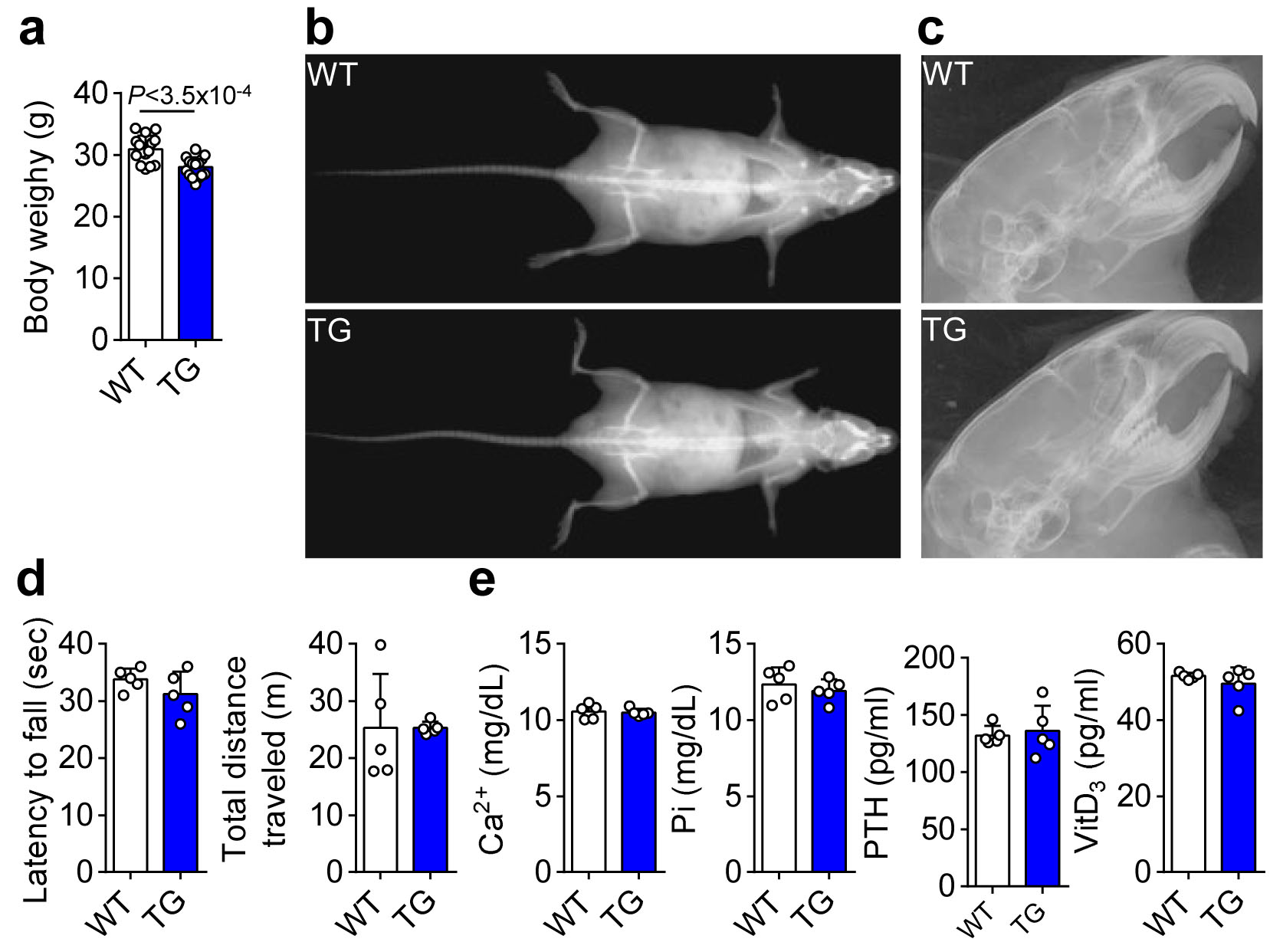


**Supplementary Figure 8** Change in body and bone appearance of *SELENOW*-overexpressing transgenic male mice at 10 weeks. **a** Comparison of body weight of wild-type (WT) and transgenic (TG) male mice (*n* = 15). **b** X-ray appearance for whole body using a Dual-Energy X-ray Absorptiometry (InAnalyzer, Medikors, Korea). **c** Examination of tooth eruption using a soft X-ray radiography. **d** Locomotor activity. Latency to fall on the rotarod with 3 cm rod diameter was recorded (left panel, *n* = 5); total distance travelled on the open field was measured using the SAMRT automated video tracking (right panel, *n* = 5). **e** Analysis of serum bone-related parameters [Ca^2+^, Pi, parathyroid hormone (PTH), vitamin D_3_ (VitD_3_)] from WT littermates and TG mice using ELISA kit (according to the manufacturer instruction, *n* = 5). Data represent mean ± SD. Statistical significance was determined by Student’s two-tailed t test.


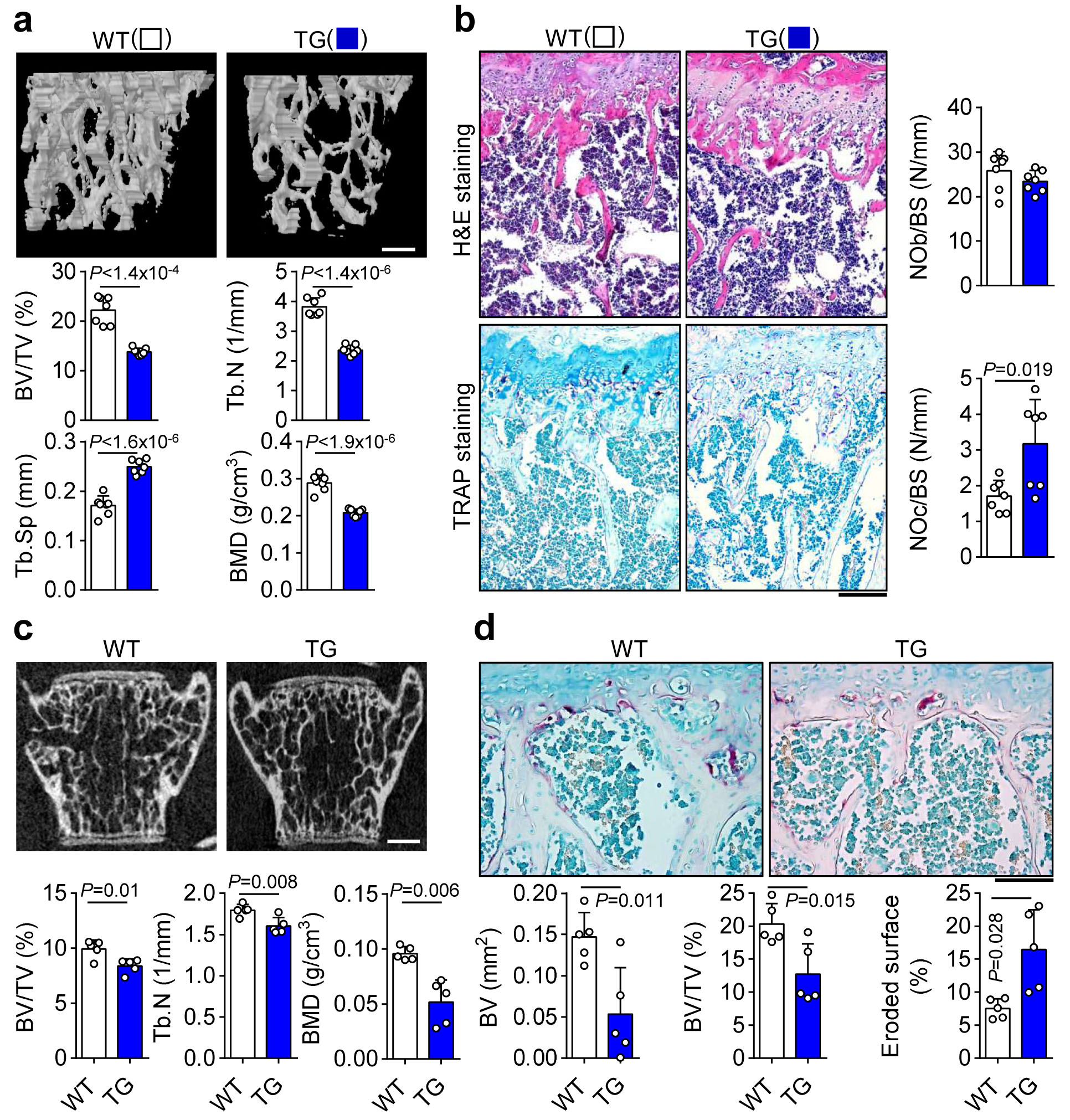


**Supplementary Figure 9** Osteoporotic phenotypes in *SELENOW*-overexpressing transgenic male mice. **a** μCT analysis of proximal tibiae from wild-type (WT) female littermates and age/sex-matched transgenic (TG) mice at 10 weeks. BV/TV, trabecular bone volume per tissue volume; Tb.N, trabecular bone number; Tb.Sp, trabecular separation; BMD, bone mineral density; Scale bar, 0.5 mm. **b** Accelerated osteoclast formation in trabecular bone surface of TG female mice; NOb/BS from H&E-stained sections (upper panels; NOc/BS from TRAP-stained sections (lower panels); Scale bar, 100 μm. **c** μCT analysis of trabecular bone in the lumbar vertebra (L4) from WT male littermates and *SELENOW*^−/−^ mice. BV/TV, trabecular bone volume per tissue volume; Tb.N, trabecular bone number. **d** Bone histomorphometric analysis for BV, BV/TV and eroded bone surface from TRAP-stained lumbar trabecular bone sections; Scale bar, 100 μm. Data represent mean ± SD (*n* = 7 mice per group in **a** and **b**, *n* = 5 mice per group in **c** and **d**). Statistical significance was determined by Student’s two-tailed t test.


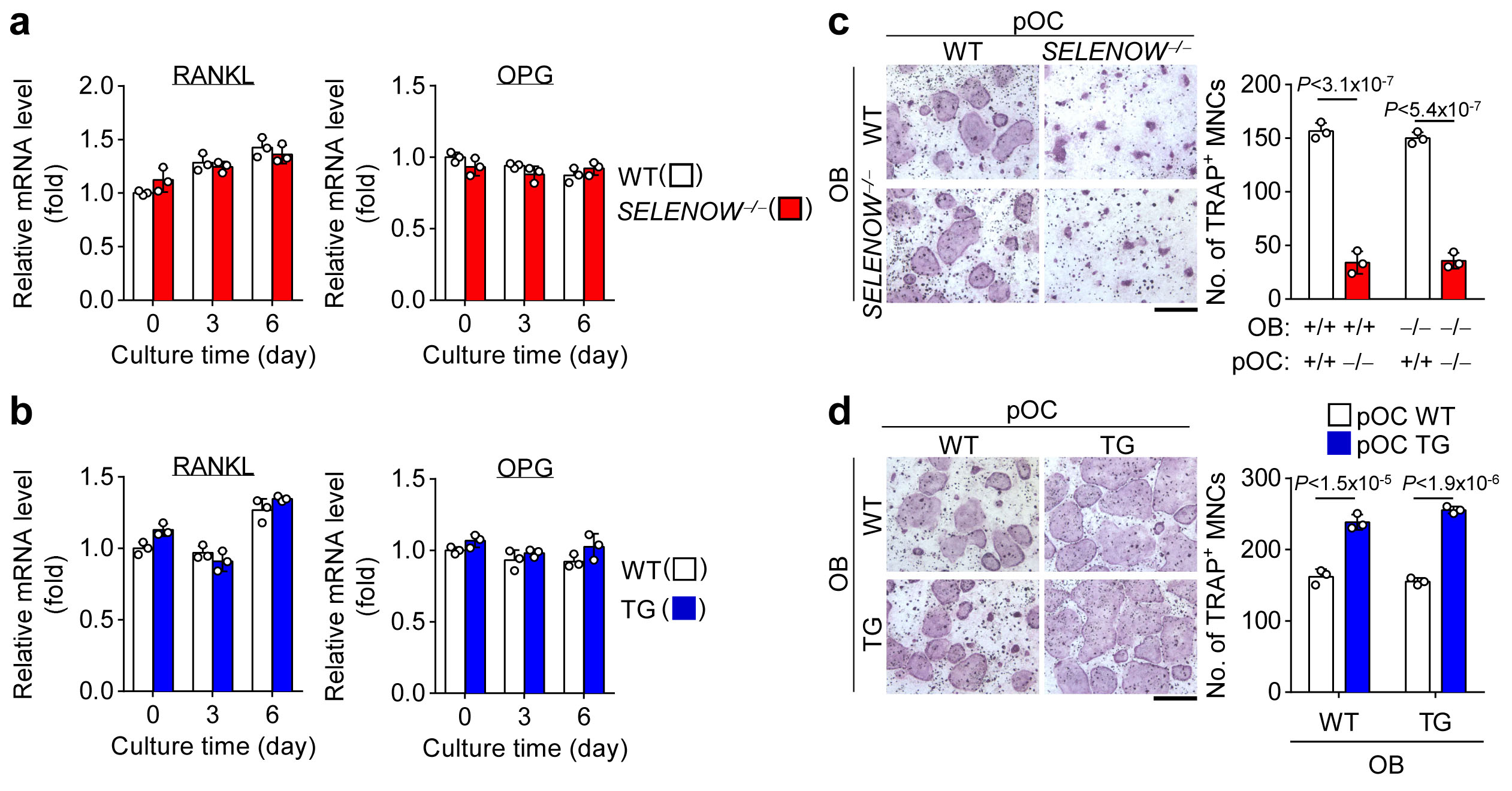


**Supplementary Figure 10** The expression level of *SELENOW* in osteoclast precursors, but not osteoblast, determines osteoclast formation. **a**, **b** Expression levels of RANKL and osteoprotegerin (OPG) in calvarial osteoblasts from *SELENOW*-deficient (*SELENOW*^−/−^) and –overexpressing transgenic (TG) mice. The calvarial osteoblasts (1 x 10^5^ cells/well in a 6-well plate) prepared from *SELENOW*^−/−^ and TG mice or WT littermates were cultured in the presence of 20 nM 1α,25-dyhydroxy vitamin D3 and 1 mM prostaglandin E2for the indicated days. The mRNA levels of RANKL and OPG were analysed using qPCR (*n* = 3). **c**, **d** Osteoclast differentiation from co-culturing osteoclast precursors and osteoblasts. Bone marrow-derived osteoclast precursors (pOC; 1 x 10^5^ cells/well in a 48-well plate) from WT (+/+) and *SELENOW*^−/−^ (−/−) mice were co-cultured with calvarial osteoblast cells (OB; 1 x 10^4^ cells/well) from WT (+/+) and *SELENOW*^−/−^(−/−) mice for 12 days. Osteoclast formation was assessed by counting TRAP-positive multinucleated cells having more than 10 nuclei (**c,** *n* = 3). The co-culture system was also applied to osteoclast precursors and osteoblasts isolated from TG mice (**d,** *n* = 3). Scale bars, 100 μm. Data represent mean ± SD of triplicate samples. One-way ANOVA was performed followed by Turkey’s test (**c** and **d**).


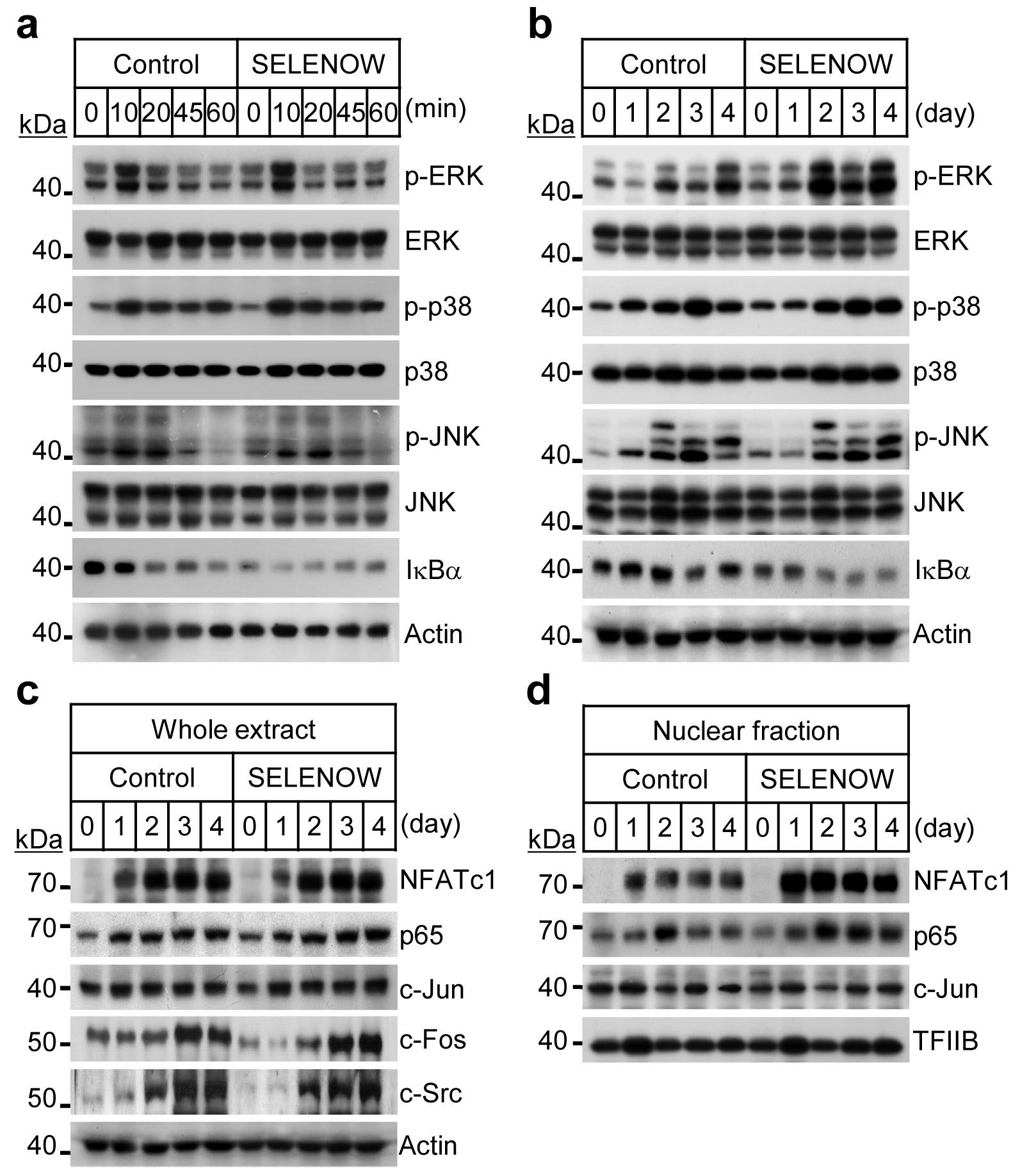


**Supplementary Figure 11** NF-κB and NFATc1 activation by ectopic expression of *SELENOW*. **a, b** Accelerated inhibitor of NF-κB (IκBα) degradation by SELENOW. Osteoclast precursors were transduced with a retrovirus harbouring *SELENOW* and selected with puromycin for 2 days in the presence of M-CSF followed by incubation with RANKL. The extent of MAPK (ERK, p38, and JNK) activation and IκBα degradation in the immediate (**a**) and delayed (**b**) response to RANKL was determined by immunoblotting. **c, d** Efficient nuclear translocation of NF-κB and NFATc1 induced by SELENOW. Osteoclast precursors were infected with a retrovirus harbouring *SELENOW* and then induced to differentiate into osteoclasts with M-CSF and RANKL; whole cell extracts (**c**) and nuclear fractions (**d**) were prepared at indicated time points, and the levels of the transcription factors NFATc1, NF-κB, and AP-1 were determined by immunoblotting. Images are representative of three independent experiments.


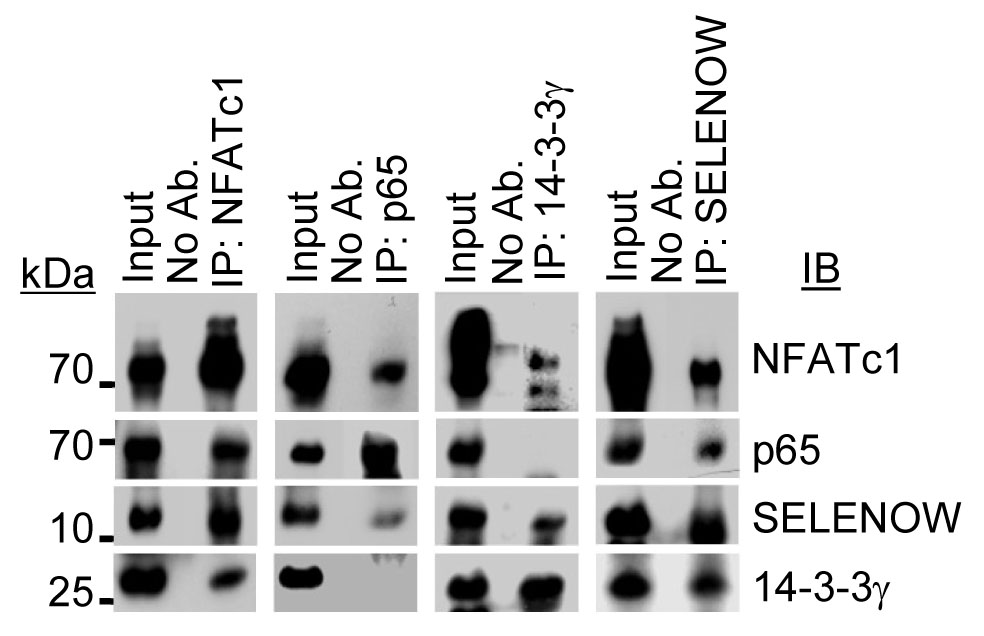


**Supplementary Figure 12** SELENOW interacts with NFATc1 or NF-κB. Osteoclast precursors infected with *SELENOW*-overexpressing retrovirus were cultured with M-CSF and RANKL for 2 days and nuclear extracts from pre-osteoclasts overexpressing SELENOW were immunoprecipitated (IP) and then immunoblotted (IB) with the indicated antibodies. Images are representative of three independent experiments.


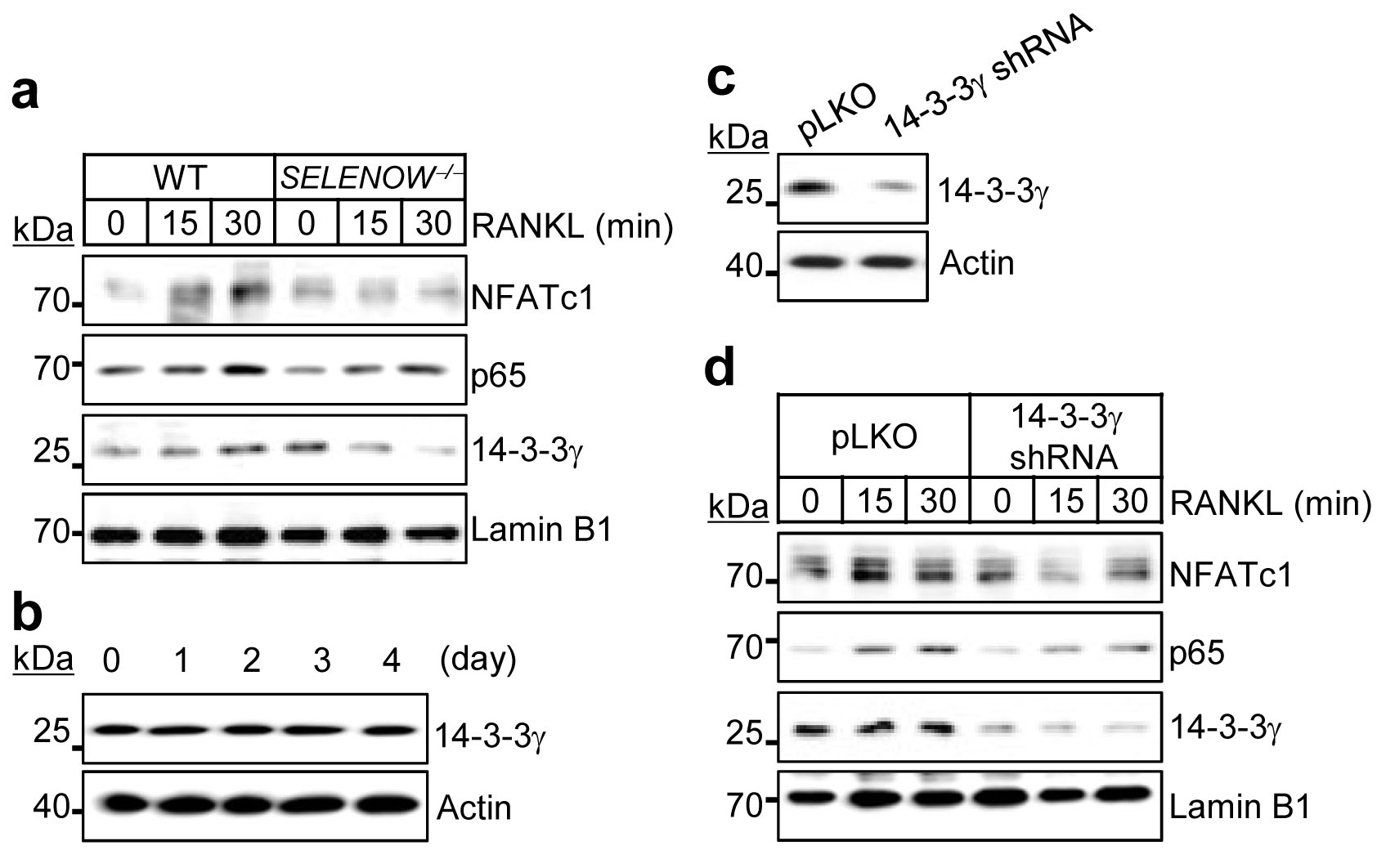


**Supplementary Figure 13** 14-3-3γ regulates nuclear translocation of NFATc1 and NF-κB p65. **a** After osteoclast precursors from wild-type (WT) and *SELENOW*^−/−^ mice were cultured with M-CSF and RANKL for 2 days to induce preosteoclasts, incubated in M-CSF- and RANKL-free condition for 3 h, and stimulated with RANKL, nuclear fractions from the cells were used for immunoblot analysis. **b** 14-3-3γ was constitutively expressed during osteoclast differentiation. **c** 14-3-3γ gene silencing using shRNA-mediated lentivirus. **d** Osteoclast precursors from WT mice were transduced with a lentivirus harbouring 14-3-3γ-targeted shRNA, treated as in (**a**), and subjected to immunoblotting. Images are representative of three independent experiments.


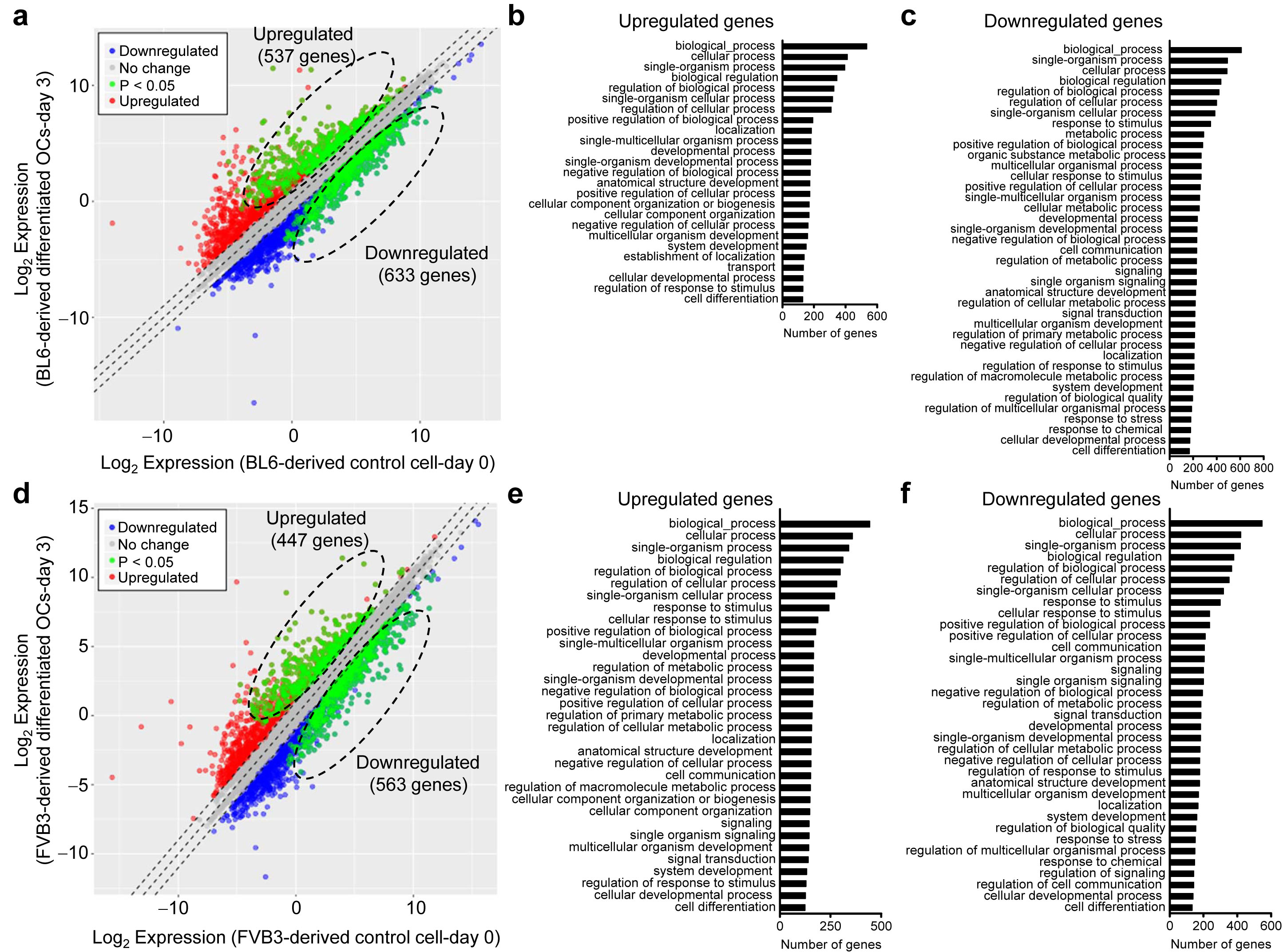


**Supplementary Figure 14** Profiles of up- and downregulated cell differentiation-related genes determined by RNA sequencing (RNA-seq) analysis of osteoclast precursors obtained from C57BL6 and FVB3 mice. **a** Scatter plot of RNA-seq expression data. Osteoclast precursors from C57BL6 wild-type mice were induced to differentiate into osteoclasts for 3 days. Based on biological processes of GO annotation categories for 23,997 genes, those with a > 2-fold increase and P value < 0.05 before (day 0) and after (day 3) RANKL-induced osteoclast differentiation were compared. A total of 537 and 633 genes were up- and downregulated, respectively. **b, c** Up- and down-regulated genes during RANKL-induced osteoclastogenesis; 129 out of 537 upregulated genes **(b)** and 168 out of 633 downregulated genes **(c)** were considered to be associated with cell differentiation. **d-f** RNA-seq analysis of osteoclast precursors from FVB3 mice. **(d)** Scatter plot of RNA-seq expression data. A total of 447 and 563 genes were significantly up- and downregulated, respectively. Cell differentiation-related up- and downregulated genes; 123 of the 447 upregulated genes **(e)** and 133 of the 563 downregulated genes **(f)** were considered to be associated with cell differentiation.


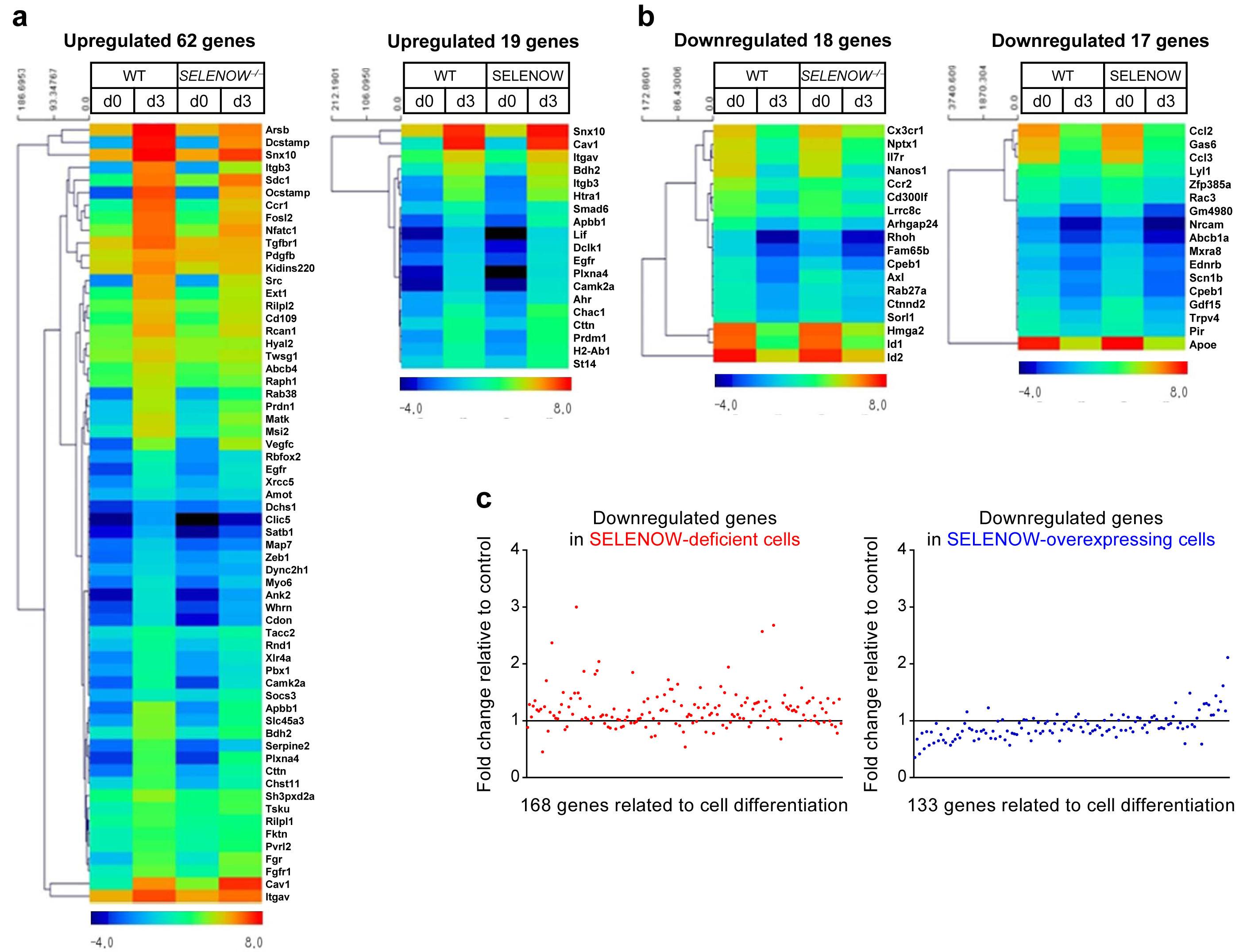


**Supplementary Figure 15** Heat map representation of significantly altered cell-differentiation-related genes and RANKL-dependent downregulated gene expression profiles in *SELENOW*-deficient and -overexpressing osteoclasts. **a** Differential expression of upregulated cell differentiation-related genes in *SELENOW*^−/−^ and *SELENOW*-overexpressing transgenic osteoclasts as compared to wild-type (WT) osteoclasts. The fold induction of upregulated genes related to cell differentiation in WT C57BL6 (129 genes) and FVB3 (123 genes) osteoclasts (as shown in Fig. 5) was set to 1, and genes showing ≥ 1.5-fold decrease or increase in *SELENOW*^−/−^ and *SELENOW*-overexpressing osteoclasts were analysed and are shown as a heat map with a pseudo-color scale. **b** Expression of downregulated cell differentiation-related genes in *SELENOW*^−/−^ and *SELENOW*-overexpressing transgenic osteoclasts was compared to that of WT osteoclasts. The fold reduction of cell differentiation-related downregulated genes in WT C57BL6 (168 genes) and FVB3 (133 genes) osteoclasts (as shown in Supplementary Fig. 14c, f) was set to 1, and genes with a ≥ 1.5-fold decrease or increase in the *SELENOW*^−/−^ and *SELENOW*-overexpressing osteoclasts were analysed and are shown in a heat map. **c** Downregulated genes with a > 2-fold decrease and P value < 0.05 were analysed as described in the legend of Fig. 5. Among downregulated genes, 168 from C57BL6 wild-type mice-derived osteoclasts and 133 from FVB3 wild-type mouse-derived osteoclasts were cell differentiation-related (as mentioned in Supplementary Fig. 14c, f and Supplementary Fig. 15b). When relative fold reduction was compared as described in the legend of Fig. 5, relative gene reduction was increased in *SELENOW*^−/−^ cells (left panel) and decreased in *SELENOW*-overexpressing cells (right panel).
