# Supplementary material for: Selenoprotein W ensures physiological bone remodeling by preventing hyperactivity of osteoclasts
Source: Nat Commun. 2021 Apr 15;12:2258. doi: 10.1038/s41467-021-22565-7 (PMC8050258; doi:10.1038/s41467-021-22565-7)
Supplement: Supplementary file 2 — Reporting Summary [file 41467_2021_22565_MOESM2_ESM.pdf]

nature research

Corresponding author(s): Daewon Jeong

Last updated by author(s): Jan 18, 2021

Reporting Summary

Nature Research wishes to improve the reproducibility of the work that we publish. This form provides structure for consistency and transparency in reporting. For further information on Nature Research policies, see our Editorial Policies and the Editorial Policy Checklist.

Statistics

For all statistical analyses, confirm that the following items are present in the figure legend, table legend, main text, or Methods section.

n/a	Confirmed	
☐	☒	The exact sample size (n) for each experimental group/condition, given as a discrete number and unit of measurement
☐	☒	A statement on whether measurements were taken from distinct samples or whether the same sample was measured repeatedly
☐	☒	The statistical test(s) used AND whether they are one- or two-sided<br>*Only common tests should be described solely by name; describe more complex techniques in the Methods section.*
☐	☒	A description of all covariates tested
☒	☐	A description of any assumptions or corrections, such as tests of normality and adjustment for multiple comparisons
☐	☒	A full description of the statistical parameters including central tendency (e.g. means) or other basic estimates (e.g. regression coefficient) AND variation (e.g. standard deviation) or associated estimates of uncertainty (e.g. confidence intervals)
☐	☒	For null hypothesis testing, the test statistic (e.g. F, t, r) with confidence intervals, effect sizes, degrees of freedom and P value noted<br>*Give P values as exact values whenever suitable.*
☒	☐	For Bayesian analysis, information on the choice of priors and Markov chain Monte Carlo settings
☒	☐	For hierarchical and complex designs, identification of the appropriate level for tests and full reporting of outcomes
☒	☐	Estimates of effect sizes (e.g. Cohen's d, Pearson's r), indicating how they were calculated

Our web collection on statistics for biologists contains articles on many of the points above.

Software and code

Policy information about availability of computer code

Data collection	No software was used for data collection
Data analysis	GraphPad Prism software (version 5 and 7), Image-Pro Plus v.6.0 software (Media Cybernetics), TopHat-2 v.2.0.13 (http://ccb.jhu.edu/software/tophat), Cuffdiff v.2.2.0 (http://cole-trapnell-lab.github.io/cufflinks/cuffdiff/) were applied for data analysis.

For manuscripts utilizing custom algorithms or software that are central to the research but not yet described in published literature, software must be made available to editors and reviewers. We strongly encourage code deposition in a community repository (e.g. GitHub). See the Nature Research guidelines for submitting code & software for further information.

Data

Policy information about availability of data

All manuscripts must include a data availability statement. This statement should provide the following information, where applicable:

- Accession codes, unique identifiers, or web links for publicly available datasets
- A list of figures that have associated raw data
- A description of any restrictions on data availability

All data supporting the findings are presented within this paper and its supplementary information. Additional data that support the findings of this study are available from the corresponding author upon reasonable request.

Field-specific reporting

Please select the one below that is the best fit for your research. If you are not sure, read the appropriate sections before making your selection.

☒ Life sciences ☐ Behavioural & social sciences ☐ Ecological, evolutionary & environmental sciences

For a reference copy of the document with all sections, see nature.com/documents/nr-reporting-summary-flat.pdf

Life sciences study design

All studies must disclose on these points even when the disclosure is negative.

Sample size	No statistical method was used to predetermine sample size. The sample size used for each experiment is indicated at the corresponding figure legend in the manuscript.
Data exclusions	No data were excluded from the analyses.
Replication	Experiments were repeated independently at least three time with similar results. The in vitro studies were performed in triplicate as indicated in the manuscript.
Randomization	Mice were randomized based on sex, age and weight. Age and gender-matched animals were used in all the experiments. In in vitro experiments , groups were allocated randomly.
Blinding	Blinding was not possible for most in vitro experiments as group allocation and treatment was administered by the researcher collecting the data. Where possible, researchers were blinded during data analysis. In most in vivo experiments, the investigators were unaware of the genotype of the experimental groups.

Reporting for specific materials, systems and methods

We require information from authors about some types of materials, experimental systems and methods used in many studies. Here, indicate whether each material, system or method listed is relevant to your study. If you are not sure if a list item applies to your research, read the appropriate section before selecting a response.

Materials & experimental systems

n/a	Involved in the study	
☐	☒	Antibodies
☐	☒	Eukaryotic cell lines
☒	☐	Palaeontology and archaeology
☐	☒	Animals and other organisms
☒	☐	Human research participants
☒	☐	Clinical data
☒	☐	Dual use research of concern

Methods

n/a	Involved in the study	
☒	☐	ChIP-seq
☒	☐	Flow cytometry
☒	☐	MRI-based neuroimaging

Antibodies

Antibodies used

Antibodies used for this study:
anti-SELENOW antibody (Novus Biologicals, NBP1-49599, rabbit polyclonal, 1:1000),
anti-β-Actin antibody (Santa Cruz Biotechnology, sc-47778, mouse monoclonal, 1:1000),
anti-NFATc1 antibody (Santa Cruz Biotechnology, sc-7294, mouse monoclonal, 1:1000),
anti-p65 antibody (Santa Cruz Biotechnology, sc-372, mouse monoclonal, 1:1000),
anti-14-3-3 gamma antibody (Santa Cruz Biotechnology, sc-398423 mouse monoclonal, 1:1000),
anti-c-Jun antibody (Santa Cruz Biotechnology, sc-1694, rabbit polyclonal, 1:1000),
anti-c-Fos antibody (Cell Signaling, #4384, rabbit polyclonal, 1:1000),
anti-6x-His Tag antibody (Thermo Scientific, MA1-21315, mouse monoclonal, 1:1000),
anti-c-Src antibody (Santa Cruz Biotechnology, sc-8056, rabbit polyclonal, 1:1000),
anti-p-ERK antibody (Cell Signaling, #9101, rabbit polyclonal, 1:1000),
anti-ERK antibody (Cell Signaling, #9102, rabbit polyclonal, 1:1000),
anti-p-JNK antibody (Cell Signaling, #9251, rabbit polyclonal, 1:1000),
anti-JNK antibody (Cell Signaling, #9252, rabbit polyclonal, 1:1000),
anti-p-p38 antibody (Cell Signaling, #9211, rabbit polyclonal, 1:1000),
anti-p38 antibody (Cell Signaling, #9212, rabbit polyclonal, 1:1000),
anti-GAPDH antibody (Abcam, ab8245, mouse monoclonal, 1:2000).

Validation	All antibodies were validated by the supplier as shown in the websites and were checked in the lab by Western blotting on cell lysate.<br>https://www.novusbio.com/products/selenoprotein-w-antibody_nbp1-49599<br>https://www.scbt.com/ko/p/beta-actin-antibody-c4<br>https://www.scbt.com/ko/p/nfatc1-antibody-7a6<br>https://www.scbt.com/ko/p/nfkappab-p65-antibody-c-20<br>https://www.scbt.com/ko/p/14-3-3-gamma-antibody-d-6<br>https://www.scbt.com/ko/p/c-jun-antibody-h-79<br>https://www.cellsignal.com/products/primary-antibodies/c-fos-antibody/4384<br>https://www.thermofisher.com/antibody/product/6x-His-Tag-Antibody-clone-HIS-H8-Monoclonal/MA1-21315<br>https://www.scbt.com/ko/p/c-src-antibody-b-12<br>https://www.cellsignal.com/products/primary-antibodies/phospho-p44-42-mapk-erk1-2-thr202-tyr204-antibody/9101<br>https://www.cellsignal.com/products/primary-antibodies/p44-42-mapk-erk1-2-antibody/9102<br>https://www.cellsignal.com/products/primary-antibodies/phospho-sapk-jnk-thr183-tyr185-antibody/9251<br>https://www.cellsignal.com/products/primary-antibodies/sapk-jnk-antibody/9252<br>https://www.cellsignal.com/products/primary-antibodies/phospho-p38-mapk-thr180-tyr182-antibody/9211<br>https://www.cellsignal.com/products/primary-antibodies/p38-mapk-antibody/9212<br>https://www.abcam.com/gapdh-antibody-6c5-loading-control-ab8245.html

Eukaryotic cell lines

Policy information about cell lines

Cell line source(s)	HEK293T (ATCC, #CRL-11268)), RAW264.7 (ATCC, TIB-71), Plat-E (Cell Biolabs)
Authentication	Cells were authenticated by the supplier. Virus packaging and transgene expression were successful. No additional authentication was performed.
Mycoplasma contamination	All cell lines tested negative for mycoplasma contamination.
Commonly misidentified lines (See ICLAC register)	No commonly misidentified cell lines were used.

Animals and other organisms

Policy information about studies involving animals; ARRIVE guidelines recommended for reporting animal research

Laboratory animals	Mouse: C57BL/6J (Central lab animal, Seoul, Korea)<br>Mouse: FVB3 (Central lab animal, Seoul, Korea)<br>Mouse: LysM-cre (Jackson Laboratory, #004781)<br>Mouse: Floxed conditional SELENOW(tm1c) allele in the C57BL/6 background (EMMA, ID EM:09166)<br>For preparation of bone marrow-derived macrophages, the femur and tibia of 6-week-old male C57BL/6J mice (Central lab animal, Seoul, Korea) were used. For preparation of liver-derived macrophages, day 14.5 to 16.5 TRAF6−/− C57BL/6J embryos (Central lab animal, Seoul, Korea) was used. For preparation of primary osteoblast precursors, the calvarial bone of newborn C57BL6 mice were used. Mice were group housed (4/cage) in a humidity and temperature-controlled room with 12h dark/light cycle in a facility with automated temperature, humidity, and light cycle control with free access to food and water.
Wild animals	No wild animals were used in the study.
Field-collected samples	Study did not involve field-collected samples.
Ethics oversight	The animal protocol and experimental procedures were approved by the Institutional Animal Care and Use Committee of Yeungnam University College of Medicine.

Note that full information on the approval of the study protocol must also be provided in the manuscript.
